# Supplementary material for: Hypoperfusion states could increase the risk of non-arteritic anterior ischemic optic neuropathy
Source: PLoS One. 2024 Nov 25;19(11):e0313098. doi: 10.1371/journal.pone.0313098 (PMC11588264; doi:10.1371/journal.pone.0313098)
Supplement: S1 Table — List of comorbidities used for analysis. (DOCX) [file pone.0313098.s001.docx]

Supplementary Table 1: List of ICD-9 Codes

| **Characteristic** | | **ICD-9 Diagnosis/ Procedure/ ATC-5 Code** |
| --- | --- | --- |
| **Hypoperfusion states** | |  |
| Reduced systemic vascular resistance | Sepsis | 995.9x |
|  | Septic shock | 782.52 |
|  | Pyelonephritis | 590.x |
|  | Peritonitis | 567.x |
|  | Septic arthritis | 711.0x, 711.9x |
|  | Necrotizing fasciitis | 728.86 |
|  | Pneumonia | 481.x, 482.x, 483.x, 484.x, 486.x, 507.x, 510.x, 511.x, 513.x |
|  | Cholecystitis | 574.0x, 574.1x, 574.3x, 574.4x, 574.6x, 574.7x, 574.8x, 575.0, 575.1x, 575.2 |
|  | Cholangitis | 576.1 |
|  | Anaphylactic shock | 995.0 |
|  | Vasopressor support | C05AX06, C01CA07, C01CA24, C01CA04, C01CA03, H01BA01, H01BA02 |
| Cardiac dysfunction | Cardiac arrest | 427.5, Z9960 |
|  | Myocardial infarction | 410.x, 411.x, 412.x, Z88.57 |
|  | Acute pulmonary edema | 518.4 |
|  | Pulmonary embolism | 415.1x, 673.x |
|  | Cardiogenic shock | 785.51 |
| Hypovolemia | GI bleeding | 578.x, 456.0, Z44.4x |
|  | Trauma | 860.x-869.x, 959.0x, 959.1x, 959.2, 959.3, 959.6, 959.7, 959.8, 959.9 |
|  | Hypotension | 458.x |
|  | Hemodialysis | Z39.95 |
|  | Major surgery | CABG: Z36.1x  ECMO: Z39.6x  Valvuloplasty: Z39.6x  Colectomy: Z35.1x, Z35.2x  Gastrectomy: Z43.5, Z43.6, Z43.7, Z43.8x, Z43.9x, Z44.3x  Cholecystectomy: Z51.2x  ERCP: Z51.10  Spine surgeries: Z81.3x  Femur surgery: Z79.05, Z79.15, Z79.25, Z79.35, Z79.45, Z79.55, Z79.65, Z79.95 |
| Others | Pneumothorax | 512.x, 860.x |
|  | Syncope | 780.2 |
|  | Aortic dissection | 441.x |
|  | Shock | 785.5x, 958.4x, 995.0, 995.4, 995.6x, 999.4x, 999.5x |
| **Systemic comorbidities** | |  |
| Liver disease | | 571.2, 571.4-571.6, 456.0-456.21, 572.2-572.8 |
| Diabetes mellitus | | 250.x |
| Renal disease | | 403.01, 403.11, 403.91, 404.02, 404.03, 404.12, 404.13, 404.92, 404.93, 582.x, 583.0-583.7, 585.x, 586.x, 588.0 |
| Malignancy | | 140.x-172.x, 174.x-195.8, 200.x-208.x, 238.6, 196.x-199.x |
| Chronic pulmonary disease | | 416.8, 416.9, 490.x-505.x, 506.4, 508.1, 508.8 |
| Myocardial infarction | | 410.x, 411.x, 412.x, Z88.57 |
| Congestive heart failure | | 398.91, 402.01, 402.11, 402.91, 404.01, 404.03, 404.11, 404.13, 404.91, 404.93, 425.4-425.9, 428.x |
| Dementia | | 290.x, 294.1, 331.2 |
| Non-arteritic anterior ischemic optic neuropathy | | 377.41 |
| Cerebrovascular disease | | 430.x-438.x, 362.34 |

GI, gastrointestinal; CABG, coronary artery bypass graft; ECMO, extracorporeal membrane oxygenation; ERCP, endoscopic retrograde cholangiopancreatography
