# Supplementary material for: Hypoperfusion states could increase the risk of non-arteritic anterior ischemic optic neuropathy
Source: PLoS One. 2024 Nov 25;19(11):e0313098. doi: 10.1371/journal.pone.0313098 (PMC11588264; doi:10.1371/journal.pone.0313098)
Supplement: S2 Table — Underlying comorbidities and demographic data of patients not included in the matching process. (DOCX) [file pone.0313098.s002.docx]

Supplementary Table 2: Patient characteristics

| **Characteristic** | **NAION**  N = 1,374 | **Controls**  N = 5,496 | **p-value** |
| --- | --- | --- | --- |
| Age, years |  |  | > 0.9 |
| Mean ± SD | 67 ± 13 | 67 ± 13 |  |
| Median (IQR) | 68 (59, 76) | 68 (59,76) |  |
| Range | 4-100 | 4-100 |  |
| Male sex, n (%) | 783 (57) | 3,132 (57) | > 0.9 |
| Ethnicity, n (%) |  |  | <0.001 |
| Jewish | 1,081 (79) | 4,548 (83) |  |
| Arab | 260 (19) | 789 (14) |  |
| Other | 33 (2.4) | 159 (2.9) |  |
| Socioeconomic status, n (%) |  |  | 0.8 |
| High | 265 (19) | 996 (18) |  |
| Medium | 822 (60) | 3,338 (61) |  |
| Low | 223 (16) | 892 (16) |  |
| Systemic comorbidities, n (%) |  |  |  |
| Liver disease | 126 (9.2) | 480 (8.7) | 0.6 |
| Diabetes mellitus | 594 (43) | 2,366 (43) | >0.9 |
| Renal disease | 204 (15) | 808 (15) | 0.9 |
| Malignancy | 157 (11) | 616 (11) | 0.8 |
| Chronic pulmonary disease | 357 (26) | 1,472 (26) | >0.9 |
| Myocardial infarction | 145 (11) | 562 (10) | 0.7 |
| Congestive heart failure | 154 (11) | 523 (9.5) | 0.064 |
| Dementia | 73 (5.3) | 327 (5.9) | 0.4 |

NAION, Non-arteritic anterior ischemic optic neuropathy; IQR, interquartile ratio
