# Supplementary material for: Hypoperfusion states could increase the risk of non-arteritic anterior ischemic optic neuropathy
Source: PLoS One. 2024 Nov 25;19(11):e0313098. doi: 10.1371/journal.pone.0313098 (PMC11588264; doi:10.1371/journal.pone.0313098)
Supplement: Attachment — Submitted filename: Response to reviewers.docx [file pone.0313098.s004.docx]

### May 18, 2024

### Emily Chenette, PhD

### Editor in Chief

### PLOS ONE

Dear Professor Chenette,

I would like to thank the reviewers for their hard effort and helpful comments. I believe our paper is better thanks to their input, and I am happy to submit the revised manuscript.

Below are attached the reviewers comments and the corresponding responses.

### Sincerely,

### Erez Tsumi, MD, MHA

### Corresponding author

Reviewer comments:

Title (line1): Consider changing to "hypoperfusion states could increase risk of Non-arteritic Anterior Ischemic Optic Neuropathy"

Response: Done

Abstract (line 30): SVR—please spell out even in abstract

Response: Done

Methods (line 75-76): *Only individuals who had been members of CHS for at least one year were included; ensuring a reliable and consistent study population.—*change to comma instead of semicolon.

Response: Done

Discussion (line 175): *and similarly a central retinal artery occlusion is considered a CVA equivalent.* However, CRAO is a completely different mechanism for stroke, presumed embolic, not stenotic. Please clarify.

Response: comment added- “Although NAION does not constitute an embolic event, the relation of retinal tissue to brain tissue is well established, and therefore an association between NAION and cardiac dysfunction is reasonable”

Discussion (line 206): *We found that patients diagnosed with NAION were 6.67 times more*

*207 likely to have had the diagnosis of cholecystitis in the month preceding the diagnosis.*

*208 Furthermore, pneumonia was also found to be associated with NAION, and patients*

*209 diagnosed with NAION were 4.5 times more likely to have been diagnosed with*

*210 pneumonia in the month preceding the diagnosis*.

I don’t follow how SVR is synonymous/always results from cholecystitis or pneumonia. Given this audience, perhaps make that link a little clearer.

Response: sentence added to clarify- “Major surgery and infection can lead to reduced SVR, and though not documented it is reasonable to assume that at least some of the patients in these groups did experience an episode reduced SVR to a certain extent”.

Discussion (line 210): *Our findings support those of Weger*

*211 et al., who investigated 71 patients and reported a 2.5-fold increase in the prevalence*

*212 of IgG antibodies for Chlamydia pneumoniae in NAION patients compared to the*

*213 control group [17].* I don’t understand why this sentence is in your paper. What is the presumed connection between chlamydia infection and NAION and would that mean a connection to sepsis? To SVR? Please eliminate or clarify further.

Response: added clarification with sentence: “which further demonstrates the possible relation of an infection to NAION, presumably through the hypoperfusion caused by reduced SVR”

Discussion (line 226): *CVA is associated with substantial*

*225 hemodynamic alterations, characterized by marked fluctuations in blood pressure that*

*226 can result in hypotension and hypoperfusion. These alterations can occur through*

*227 various mechanisms, including cerebral autoregulation [20] and sedation during*

*228 endovascular stroke interventions [21].*

This type of pathophysiologic explanation/reasoning is good and similar reasoning should be used to explain the connection of your potential associations in earlier explanations of sepsis, pneumonia, and other diagnoses.

Response: sentences added for above comments
