# Supplementary material for: Hypoperfusion states could increase the risk of non-arteritic anterior ischemic optic neuropathy
Source: PLoS One. 2024 Nov 25;19(11):e0313098. doi: 10.1371/journal.pone.0313098 (PMC11588264; doi:10.1371/journal.pone.0313098)
Supplement: Attachment — Submitted filename: Rebuttal letter 290824.docx [file pone.0313098.s005.docx]

### August 29, 2024

### Emily Chenette, PhD

### Editor in Chief

### PLOS ONE

Dear Professor Chenette,

I would once again like to thank the reviewers for their hard effort and helpful comments. I believe our paper is better thanks to their input, and I am happy to re-submit the revised manuscript, according to the comments we have received.

Below are attached the reviewers’ new comments and the corresponding responses.

### Sincerely,

### Erez Tsumi, MD, MHA

### Corresponding author

Reviewer comments:

Reviewer #1:

Agree with the revisions and only one suggestion: change running title from Short title: Risk factors for non-arteritic anterior ischemic optic neuropathy to Short title: Hypoperfusion States in non-arteritic anterior ischemic optic neuropathy

Response:

Done, line 14

Reviewer #3:

Thank you for submitting the results of a large cohort of NAION patients.There are a few glaring flaws in the claims made in the paper.
1.This is not the first paper to address the association of the NAION to ischemic heart diseases.Authors have referenced Dr Hayrehs paper on the systemic diseases and the NAION(REF NO.8) that states" Also, middle-aged and elderly patients showed a significantly higher prevalence of ischemic heart disease (P < .01)"
The authors have chosen to selectively quote the articles details mentioning only the predisposing factors like HT,DM etc,at another place Dr Hayreh has clearly defined the predisposing factors and the precipitating factors in the evolution of the NAION.The hypoperfusion states of shock,be it the cardiogenic,hypovolemic etc are mentioned in the precipitating factors.
Hayreh, S S. Ischaemic optic neuropathy.. Indian Journal of Ophthalmology 48(3):p 171-194, Jul–Sep 2000.
The above paper clearly defines the following

Perfusion pressure = Mean BP minus intraocular pressure (IOP).

Mean BP = Diastolic BP + 1/3 (systolic minus diastolic BP).
Resistance to blood flow
This depends upon the state and calibre of the vessels supplying the ONH, which in turn are influenced by the following:
.....which includes systemic hypotension

Dr SS Hayreh has studied and extensively published about the pathophysiology of NAION.His work had already indicated what your study has observed in a large cohort.
I suggest that the introduction and the discussion be revised to present in a fair manner.If possible get the details of posterior and anterior ischemic optic neuropathy in the group,as the hypoperfusion associated with hypovolemia,is most likely to cause posterior ischemic optic neuropathy.

Response:

We acknowledge that (to paraphrase off Isacc Newton) we stand on the shoulders of giants such as Dr SS Hayreh and that some of our findings were previously reported. The strength of our findings is in the large size of cohort, which can both help reach new conclusions and establish the validity of existing knowledge.

We have rephrased the relevant paragraph (lines 65-69) as well as rephrased the following sentence (lines 71-72).

Additionally- a comment was added in the discussion (lines 239-241).

With regards to diagnosing PION- we have decided to omit patients who were diagnosed with PION because we found that reporting of that diagnosis was inconsistent in patient’s files and did not allow a reliable large scale study. We added a comment in discussion (lines 247-250)
