# Supplementary material for: Hypoperfusion states could increase the risk of non-arteritic anterior ischemic optic neuropathy
Source: PLoS One. 2024 Nov 25;19(11):e0313098. doi: 10.1371/journal.pone.0313098 (PMC11588264; doi:10.1371/journal.pone.0313098)
Supplement: Attachment — Submitted filename: PONE hypoperfusion NAION Reviewer comments.docx [file pone.0313098.s003.docx]

Reviewer comments:

Title (line1): Consider changing to "hypoperfusion states could increase risk of Non-arteritic Anterior Ischemic Optic Neuropathy"

Abstract (line 30): SVR—please spell out even in abstract

Methods (line 75-76): *Only individuals who had been members of CHS for at least one year were included; ensuring a reliable and consistent study population.—*change to comma instead of semicolon.

Discussion (line 175): *and similarly a central retinal artery occlusion is considered a CVA equivalent.* However, CRAO is a completely different mechanism for stroke, presumed embolic, not stenotic. Please clarify.

Discussion (line 206): *We found that patients diagnosed with NAION were 6.67 times more*

*207 likely to have had the diagnosis of cholecystitis in the month preceding the diagnosis.*

*208 Furthermore, pneumonia was also found to be associated with NAION, and patients*

*209 diagnosed with NAION were 4.5 times more likely to have been diagnosed with*

*210 pneumonia in the month preceding the diagnosis*.

I don’t follow how SVR is synonymous/always results from cholecystitis or pneumonia. Given this audience, perhaps make that link a little clearer.

Discussion (line 210): *Our findings support those of Weger*

*211 et al., who investigated 71 patients and reported a 2.5-fold increase in the prevalence*

*212 of IgG antibodies for Chlamydia pneumoniae in NAION patients compared to the*

*213 control group [17].* I don’t understand why this sentence is in your paper. What is the presumed connection between chlamydia infection and NAION and would that mean a connection to sepsis? To SVR? Please eliminate or clarify further.

Discussion (line 226): *CVA is associated with substantial*

*225 hemodynamic alterations, characterized by marked fluctuations in blood pressure that*

*226 can result in hypotension and hypoperfusion. These alterations can occur through*

*227 various mechanisms, including cerebral autoregulation [20] and sedation during*

*228 endovascular stroke interventions [21].*

This type of pathophysiologic explanation/reasoning is good and similar reasoning should be used to explain the connection of your potential associations in earlier explanations of sepsis, pneumonia, and other diagnoses.
